# Current Hospital Topics

**Published:** 1906-07-07

**Authors:** 


### CURRENT HOSPITAL TOPICS.

### Mistakes in Advertising.

It would not be difficult to give instances where hospital advertising has failed, or to state the reasons which have led to such failures. It is bad business, for instance, for a Hospital Sunday Fund to spend money in newspaper advertisements which appear in the newspapers several weeks before the day of collection. The most effective means of advertising Hospital Sunday is to concentrate all the advertisements into the week immediately preceding the day on which the sermons will be preached. In this way the co-operation of the editors and the full advantages of the advertising columns can be united, when the cash results can be made, under vigorous management, gratifying to those who have the ability and wisdom to pursue them intelligently. Intermittent purposeless advertisements in unsuitable mediums must fail, and deserve to fail. But to make an opportunity fixing public attention and to reap abundantly in results is the work of a true artist. Who that is successful as a reaper for hospitals can fail as an artist?

### Hospital Appeals and Hospital Advertisements.

There are two recognised methods of raising funds for hospitals, one by the issue of thousands of appeals or circulars, and the other by advertisements. In London, with a very few exceptions, the latter method has never been elaborated or vigorously worked. It is admitted that the first, or appeal, form is, however, nearly, if not quite, obsolete, and that the results attained by it nowadays are usually anything but satisfactory. The cheapening of postage, and the consequent and almost daily flooding of the breakfast table with numerous communications of the kind have led the public in self-defence to acquire the habit of consigning most or all such communications to the waste-paper basket on their receipt. In the United States advertising has been reduced to a fine art, and the enormous success of the *Times* Book Club, and of a good many other ventures, where advertising has been cleverly systematised, proves that nowadays that form of approaching the public can be made fruitful in cash. The *Dreadnought* Seamen's Hospital owes its financial success to-day mainly to advertising, and the late Mr. Adrian Hope, who understood advertising and had made a study of it, raised a very large sum for the Children's Hospital in this way. A few advertisements here and there and the method which includes an article on the charity with a mention of its present official or officials, when ten guineas or more is paid for an advertisement, may be good business for the publications which practise it, but must largely result in waste of money to weak charities. We are confident, however, in view of the study we have given to methods of advertising, and the knowledge we have thus acquired of the results actually secured, that the most successful beggar for hospitals in the future is likely to prove to be the man who evolves a novel scheme and has the courage to work it boldly in the cause of his charity.

### The Question of Sites.

We deal with St. George's Hospital in another column, but the question of its site is all-important at the moment, and involves a financial proposition by no means difficult to understand. From inquiries we have made we are confident that Mr. West the Treasurer is correct in maintaining that he can procure £400,000 for the site of St. George's Hospital if he is given a free hand. A new hospital of 350 beds should be capable of being erected and equipped for £175,000, so that with the cost of the new site, on a liberal estimate, the new hospital should not entail an expenditure of more than £230,000 to £250,000. This would leave a margin of £150,000 for investment, which, at 4 per cent. interest, would increase the income of St. George's Hospital by £6,000 a year. St. George's Hospital would then be a modern, up-to-date hospital, and its usefulness would be greatly increased from the circumstance that a wisely chosen site would make it immeasurably more important in the public sense from being in a locality where the new hospital would supply the needs of a large and poor population. To remain at Knightsbridge, St. George's will have to buy up further leasehold interests, the cost of which, with the necessary tinkering and patching, will not be less than £120,000. On the other hand, granted that the hospital should remain on its present site, if that site were to be cleared and a new hospital erected upon it, Mr. West estimates that an expenditure of £350,000 will be entailed; the available saleable stock of St. George's Hospital is only £204,000, leaving a deficiency of £146,000 to be raised from the public for the building fund, together with the loss of some £8,000 a year of the present income. It follows that the only alternative to removal must result in a net loss of £14,000 a year in income, and a demand on the public for a sum of money so large as to make it certain in the circumstances that it would not be subscribed. Removal to a new site should add some £6,000 a year to its present income, and give St. George's Hospital invested funds amounting to between £300,000 and £400,000. If these proposals were to be submitted to any half dozen men of business experience the decision could on the facts only be unanimous in favour of Mr. West's plan. We have little doubt therefore that his proposals will receive the hearty support of the President of St. George's Hospital and also be sustained by the authorities who regulate the disbursement of the various great Hospital Funds.

